# Exposure to Multiple Fine Particulate Matter Components and Incident Depression in the US Medicare Population

**DOI:** 10.1001/jamanetworkopen.2025.51042

**Published:** 2025-12-22

**Authors:** Yanling Deng, Hua Hao, Qiao Zhu, Yang Liu, Kyle Steenland

**Affiliations:** 1 Gangarosa Department of Environmental Health, Rollins School of Public Health, Emory University, Atlanta, Georgia

## Abstract

**Importance:**

Growing evidence suggests that long-term exposure to fine particulate matter (PM_2.5_) is associated with an increased risk of depression. However, the contributions of specific PM_2.5_ components and the modifying roles of comorbidities remain unclear.

**Objective:**

To evaluate the individual and joint associations of long-term exposure to PM_2.5_ and its major components with incident depression, and to assess whether comorbidities modify these associations.

**Design, Setting, and Participants:**

This nationwide, population-based cohort study used Medicare data from January 2000 to December 2018. Medicare beneficiaries aged 65 years or older who were continuously enrolled in the fee-for-service component of Medicare only, resided in the contiguous US during the study period, and had both Part A and Part B were included. Data were analyzed from November 2023 to October 2025.

**Exposures:**

Annual concentrations of PM_2.5_ mass and its major components, including elemental carbon, ammonium, nitrate, sulfate, soil dust (largely silicon), and organic carbon, were assessed using a spatiotemporal model.

**Main Outcomes and Measures:**

Late-life depression diagnoses were identified by Medicare claims. Cox proportional hazard models and quantile g-computation (QGC) were applied to estimate the individual and joint associations of PM_2.5_ and its major components with depression risk. Stratified analyses by various comorbidities were also applied to evaluate the potential effect size modifiers.

**Results:**

Among 23 696 223 older adults (mean [SD] age at entry, 76.0 [6.4] years; 13 081 668 female [55.2%]), 5 544 678 developed depression during follow-up. In single-pollutant models, PM_2.5_ mass (hazard ratio [HR], 1.02; 95% CI, 1.01-1.02) and 4 of 6 major components (sulfate: HR, 1.05; 95% CI, 1.04-1.06; elemental carbon: HR, 1.03; 95% CI, 1.03-1.04; soil dust: HR, 1.03; 95% CI, 1.03-1.04; and ammonium: HR, 1.01; 95% CI, 1.01-1.02) were positively associated with depression. A mixture of multiple PM_2.5_ major components had a much larger association with depression than PM_2.5_ mass itself, with each 1-quartile increase in the mixture being associated with increased depression risk (HR, 1.07; 95% CI, 1.06-1.07). The QGC further identified soil dust (weight = 0.41), sulfate (weight = 0.34), and elemental carbon (weight = 0.25) as the main contributors. Associations were more pronounced among individuals with cardiometabolic and neurologic comorbidities.

**Conclusions and Relevance:**

In this cohort study of the US Medicare population, specific PM_2.5_ components (sulfate, elemental carbon, and soil dust) were associated with increased depression risk in older adults, particularly those with preexisting comorbidities. These findings underscore the importance of targeted regulation of harmful PM_2.5_ components to protect vulnerable populations.

## Key Points

**Question:**

What are the associations of long-term exposure to multiple fine particulate matter (PM_2.5_) major components with incident depression among older adults in the US, and what roles do comorbidities play?

**Findings:**

In this cohort study of 23 696 223 older adults, long-term exposure to PM_2.5_ components, mainly due to sulfate, elemental carbon, and soil dust, was associated with increased risk of depression. The associations were more pronounced among individuals with cardiometabolic and neurologic comorbidities.

**Meaning:**

These findings suggest that regulation of specific PM_2.5_ components may help reduce depression risk, particularly among vulnerable older populations.

## Teaser

This cohort study analyzes the individual and joint associations of long-term exposure to fine particulate matter (PM_2.5_) and its major components with incident depression among Medicaid beneficiaries.

## Introduction

Depression, characterized by core symptoms such as persistent depressed mood and decreased interest, is the most common mental disorder.^1^ The Global Burden of Disease 2021 estimates that depressive disorders accounted for approximately 56.3 million disability-adjusted life years globally in 2021, making them the leading cause of disability-adjusted life years among mental health conditions.^2^ Depression in elderly people has been associated with comorbid physical disease, cognitive impairment, and premature mortality,^3,4^ causing a significant social and economic burden.^5^ It is crucial to identify modifiable risk factors for the development of depression in older adults in order to establish effective prevention strategies.

A growing body of evidence suggests that exposure to air pollution is associated with an increased risk of depression.^6,7,8,9,10^ Several systematic reviews have concluded that there is a consistent association of exposure to fine particulate matter (PM_2.5_) with a high risk of depression.^11,12,13^ PM_2.5_ is a complex mixture of multiple chemical components, including sulfate (SO_4_^2−^), nitrate (NO_3_^−^), ammonium (NH_4_^+^), elemental carbon, organic carbon, and soil dust, that arise from diverse anthropogenic and natural sources and exhibit varying toxicities.^14^ Given the complexity of PM_2.5_ and the varying toxic effects of its components on health outcomes including depression,^15,16^ identifying which components of PM_2.5_ are the most hazardous is of public health concern and will help to further provide new opportunities to curb the burden of PM_2.5_-related depression.

To date, only a few studies have examined the associations of different PM_2.5_ components with depression risk, considering both short-term^17,18,19^ and long-term exposure.^18,20,21^ Those studies have been limited by relatively small sample sizes, have been conducted exclusively outside the US, and have focused on one component at a time. In reality, individuals are simultaneously exposed to several major components of PM_2.5_, and evaluating the joint associations of these components as a whole has been increasingly common.^22,23^ To our knowledge, only 1 study^24^ has evaluated long-term exposure to PM_2.5_ component mixtures in relation to depression symptoms, and it was limited to a Chinese cohort. Moreover, while the large positive associations of various comorbidities such as hypertension, dementia, cardiovascular diseases, and diabetes with depression risk are well documented,^25,26,27,28^ it remains unclear whether these comorbidities modify the association of the major components of PM_2.5_ with depression.

To address this research gap, we conducted a national cohort study of US Medicare beneficiaries from 2000 to 2018 to evaluate the individual and joint associations of major PM_2.5_ components with incident depression. We further assessed whether these associations varied by comorbidities and demographic characteristics.

## Methods

### Study Population

This open cohort study was established using the Medicare denominator file from the Centers for Medicare & Medicaid Services (CMS), covering the period from January 2000 to December 2018, as detailed in previous studies.^9,15^ In brief, the study participants consisted of all Medicare beneficiaries aged 65 years or older who were (1) continuously enrolled in the fee-for-service component of Medicare only, (2) residing in the contiguous US between 2000 and 2018, and (3) had both Part A (for hospital care) and Part B (for outpatient care). The Medicare denominator file provided each Medicare beneficiary’s enrollment records annually, including zip code of residence, demographic characteristics (eg, race, sex, and age), the date of death (if any), Medicaid eligibility, as well as the duration of enrollment in Part A and Part B. Our research was conducted in accordance with the Strengthening the Reporting of Observational Studies in Epidemiology (STROBE) reporting guideline for cohort studies and was approved by the Emory institutional review board and the Centers for Medicare & Medicaid Services under the data use agreement. Informed consent was waived because we conducted a secondary analysis of deidentified data.

### Exposure Assessment

Superlearning and ensemble weighted averaging models with a 1-km scale in nonurban areas and a 50-m spatial scale in urban areas were used to estimate the annual mean levels for 6 major components including organic carbon, elemental carbon, soil dust, nitrate, and ammonium, and sulfate. These dual-domain resolutions were chosen to capture finer intraurban gradients and smoother regional backgrounds.^29^ Model performance was high, with test-set cross-validated *R*^2^ values ranging from 0.86 to 0.96 for total PM_2.5_ mass and its 6 major components.^29,30^ Address information was limited to zip codes, so we used average exposure for the zip code where the participant lived, estimated for each year of residence, usually combining exposure estimates across both urban and nonurban areas. The eMethods in Supplement 1 briefly provide information about exposure assessment, with further details described elsewhere.^29^

### Outcome Assessment

Depression was identified as the first occurrence of a diagnosis of depression using a validated algorithm from the Medicare Chronic Conditions Data Warehouse, based on the *International Classification of Diseases, Ninth Revision* (*ICD-9*) or *International Statistical Classification of Diseases and Related Health Problems, Tenth Revision (ICD-10) *codes from all available Medicare claims (eg, hospital inpatient and outpatient claims, home health care claims, skilled nursing facility, and carrier claims).^31^ The date of the first depression diagnosis was sourced from the Medicare Chronic Conditions Data Warehouse after beneficiaries enrolled in the Medicare fee-for-service program. Detailed codes are provided in eTable 1 in Supplement 1. To better capture depression incidence rather than prevalence, we required a 5-year clean period without depression, preceding the first diagnosis of depression. Details regarding cohort entry, time scale, and follow-up definition are provided in the eMethods in Supplement 1.

### Covariates

We considered a range of potential confounding variables that could influence the associations of long-term exposure to PM_2.5_ and its major components with depression, guided by evidence from previous studies.^7,9,21^ The detailed information for covariates is described in the eMethods in Supplement 1.

### Statistical Analysis

Stratified Cox proportional hazard models with generalized estimating equations^32^ were applied to examine the associations of long-term individual average PM_2.5_ mass and its major components (calculated as a 5-year moving average) with incident depression risk, using years of follow-up as the time scale. The generalized estimating equations framework was used to account for within-zip code correlation of residuals and provide robust variance estimates. To estimate the shape of the concentration response for the association of PM_2.5_ and its major components with depression risk, we applied penalized splines for each exposure in single-pollutant models. Quantile g-computation (QGC) was applied to explore the joint associations of the PM_2.5_ major component mixtures with incident depression risk. Compared with the weighted quantile sum approach, which is not directly applicable to survival data and assumes all exposures act in the same direction, QGC is a generalization of the weighted quantile sum approach for mixture analyses. QGC is less subject to multicollinearity issues when estimating the effect size of a simultaneous quantile increase in all components, although the estimates for each component may still suffer from multicollinearity. QGC also does not require all components to have associations in the same direction.^33^

We conducted several stratification analyses by demographics, race (Black, White, and other [American Indian or Alaskan Native, Asian, Hispanic, and unknown]), Medicaid eligibility, age, region, and various comorbidities available from the Medicare data to determine which subpopulations may be more susceptible than others. Comorbidities were evaluated as potential effect size modifiers rather than mediators because the study was designed to further characterize susceptible groups rather than to decompose potential causal pathways. In addition, several sensitivity analyses were conducted to estimate the robustness of our main results.

Further details of the statistical models are provided in the eMethods in Supplement 1. All statistical analyses were performed on the Rollins high-performance computing Cluster at Emory University using R software version 4.2.3 (R Project for Statistical Computing) and Python software version 3.12.1 (Python Software Foundation) from November 2023 to October 2025. A 2-sided *P* < .05 was considered statistically significant.

## Results

### Study Population Characteristics

Table 1 displays descriptive information on the depression cohort from 2000 to 2018. This present study included 23 696 223 participants (mean [SD] age at entry, 76.0 [6.4] years; 13 081 668 female [55.2%]), of which approximately 5 544 678 developed depression. Most of the participants were never eligible for Medicaid eligibility (20 114 980 participants [84.9%]). Of all participants, 1 733 688 (7.3%) were Black, 20 972 565 (88.5%) were White, and 989 970 (4.2%) identified as another race. Of participants with depression, 5353 (96.6%) had at least 1 comorbidity at some point during follow-up, with the majority experiencing hypertension (5 072 488 participants [91.5%]). eFigure 1 in Supplement 1 presents the zip code–level occurrences of first depression events from 2000 to 2018 per 100 000 Medicare beneficiaries across the contiguous US.

**Table 1.** Descriptive Statistics for the Study Population and Distribution of Air Pollution Among the Cohort With a 5-Year Clean Period^a^

Variables	Participants, No. (%)		
	Full cohort (N = 23 696 223)	Depression (n = 5 544 678)	No depression (n = 18 151 545)
Total person-years	145 477 746	30 190 295	115 287 451
No. of follow-up years, median (IQR)	5 (7)	5 (6)	5 (8)
Age at entry, y	75.97 (6.42)	77.05 (6.27)	75.65 (6.44)
<75	13 303 345 (56.1)	2 486 715 (44.8)	10 816 630 (59.6)
≥75	10 392 878 (43.9)	3 057 963 (55.2)	7 334 915 (40.4)
Sex			
Male	10 614 555 (44.8)	2 013 420 (36.3)	8 601 135 (47.4)
Female	13 081 668 (55.2)	3 531 258 (63.7)	9 550 410 (52.6)
Race			
Black	1 733 688 (7.3)	301 840 (5.4)	1 431 848 (7.9)
White	20 972 565 (88.5)	5 072 103 (91.5)	15 900 462 (87.6)
Other^b^	989 970 (4.2)	170 735 (3.1)	819 235 (4.5)
Medicaid eligibility			
Ineligible	20 114 980 (84.9)	4 512 934 (81.4)	15 602 046 (86.0)
Ever eligible	3 581 243 (15.1)	1 031 744 (18.6)	2 549 499 (14.0)
Region			
Midwest	6 160 160 (26.0)	1 432 369 (25.8)	4 727 791 (26.0)
Northeast	4 309 655 (18.2)	1 064 364 (19.2)	3 245 291 (17.9)
Southeast	7 546 390 (31.8)	1 785 897 (32.2)	5 760 493 (31.7)
Southwest	2 458 787 (10.4)	589 594 (10.6)	1 869 193 (10.3)
West	3 221 231 (13.6)	672 454 (12.1)	2 548 777 (14.0)
Comorbidities	21 617 270 (91.2)	5 353 809 (96.6)	16 263 461 (89.6)
Alzheimer disease	2 920 148 (12.3)	1 243 917 (22.4)	1 676 231 (9.2)
Dementia	5 869 632 (24.8)	2 097 638 (37.8)	3 771 994 (20.8)
Chronic obstructive pulmonary disease	7 791 300 (32.9)	2 134 817 (38.5)	5 656 483 (31.2)
Stroke	5 134 577 (21.7)	1 544 006 (27.8)	3 590 571 (19.8)
Congestive heart failure	9 387 179 (39.6)	2 536 877 (45.8)	6 850 302 (37.7)
Diabetes	8 957 476 (37.8)	2 273 925 (41.0)	6 683 551 (36.8)
Hypertension	20 310 130 (85.7)	5 072 488 (91.5)	15 237 642 (83.9)
Lung cancer	929 894 (3.9)	178 336 (3.2)	751 558 (4.1)
No comorbidities^c^	2 078 953 (8.8)	190 869 (3.4)	1 888 084 (10.4)
Air pollutants, median (IQR), μg/m^3^^d^			
Elemental carbon	0.52 (0.26)	0.55 (0.26)	0.51 (0.26)
Ammonium	0.95 (0.75)	1.08 (0.73)	0.92 (0.73)
Sulfate	2.39 (1.73)	2.83 (1.59)	2.27 (1.71)
Nitrate	0.97 (0.92)	1.01 (0.97)	0.96 (0.91)
Soil dust	0.60 (0.30)	0.62 (0.29)	0.60 (0.30)
Organic carbon	1.76 (0.66)	1.82 (0.67)	1.75 (0.66)
Fine particulate matter (PM_2.5_) mass	10.17 (3.82)	10.83 (3.82)	10.0 (3.77)
Other environmental factors^e^			
Temperature, mean (SD), °C	14.4 (4.6)	14.5 (4.6)	14.4 (4.6)
Relative humidity, mean (SD), %	69.1 (11.0)	69.4 (10.7)	69.1 (11.1)
Smoke rate, mean (SD), %	46.4 (7.4)	46.7 (7.3)	46.3 (7.4)
No. of hospitals, mean (SD)	10.0 (17.6)	10.3 (17.9)	9.9 (17.6)
Population density, mean (SD), people per km^2^	966.6 (2737.0)	988.0 (2739.3)	961.0 (2736.4)
Black race, mean (SD), %	10.4 (16.6)	10.1 (15.8)	10.5 (16.8)
Median household income, mean (SD), $1000	55.7 (23.1)	54.1 (22.0)	56.1 (23.3)
Renting house or apartment, mean (SD), %	30.8 (14.5)	30.7 (14.5)	30.9 (14.5)
Below poverty level, mean (SD), %	12.8 (7.5)	12.6 (7.4)	12.9 (7.6)
Not graduated from high school, mean (SD), %	14.2 (8.3)	14.8 (8.4)	14.1 (8.3)

^a^ Table 1 consists of complete data without missing variables.

^b^ Other included American Indian, or Alaskan Native, Asian, Hispanic, and unknown. Further divisions of race were not possible owing to the structure of the data.

^c^ None of the listed comorbidities.

^d^ Exposure was treated as 5-year-before window moving means of air pollutants.

^e^ Annual mean levels.

### Distribution of PM_2.5_ Major Components

The 5-year window mean (SD) level of PM_2.5_ was 10.19 (2.86) μg/m^3^. Among the 6 major components, sulfate had highest annual mean levels, followed by organic carbon, nitrate, ammonium, soil dust, and elemental carbon (eTable 2 in Supplement 1). The annual IQRs were 3.82 μg/m^3^ for PM_2.5_ total mass, 0.26 μg/m^3 ^for elemental carbon, 0.75 μg/m^3^ for ammonium, 1.73 μg/m^3^ for sulfate, 0.92 μg/m^3 ^for nitrate, 0.30 μg/m^3 ^for soil dust, and 0.66 μg/m^3 ^for organic carbon (Table 1). eFigure 2 in Supplement 1 illustrates the correlation matrix for PM_2.5_ mass and the 6 primary components. All PM_2.5_ major components except for soil dust demonstrated moderate to strong correlations with PM_2.5_ mass, with Pearson correlation coefficients between 0.56 and 0.86. eFigure 3 in Supplement 1 shows the pie chart of the mean chemical composition of PM_2.5_ mass concentrations. eFigure 4 in Supplement 1 presents the spatial distributions of major PM_2.5_ components across the US from 2000 to 2018.

### Health Effect Size Estimates

#### Single-Pollutant Model Based on the Cox Proportional Hazard Models

Table 2 displays the results from single-pollutant Cox proportional hazard models, where long-term exposure to PM_2.5_ mass and 4 of its 6 major components showed a significant association with an increased risk of depression. Per IQR increase in the 5-year average concentrations of these exposures, the largest hazard of depression was for sulfate (hazard ratio [HR], 1.05; 95% CI, 1.04-1.06), followed by elemental carbon (HR, 1.03; 95% CI, 1.03-1.04) and soil dust (HR, 1.03; 95% CI, 1.03-1.04), PM_2.5_ mass (HR, 1.02; 95% CI, 1.01-1.02), and ammonium (HR, 1.01; 95% CI, 1.01-1.02).

**Table 2.** Hazard of Depression per IQR Increase in PM_2.5 _Major Components From Single-Pollutant Models Among the Cohort With a 5-Year Clean Period^a^

Variable^b^	Depression, HR (95% CI)^c^
Depression events/total person-years	5 544 678/145 477 746
Elemental carbon	1.03 (1.03-1.04)
Ammonium	1.01 (1.01-1.02)
Sulfate	1.05 (1.04-1.06)
Nitrate	0.99 (0.99-1.00)
Soil dust	1.03 (1.03-1.04)
Organic carbon	1.00 (1.00-1.01)
PM_2.5_ mass	1.02 (1.01-1.02)

Abbreviations: HR, hazard ratio; PM_2.5_, fine particulate matter.

^a^ This analysis was performed with a full cohort including 23 696 223 Medicare beneficiaries with 145 477 746 person-years. All models were adjusted for individual-level characteristics (age, sex, race, and Medicaid eligibility), neighborhood-level socioeconomic indicators (percentage of the population below the poverty level, population density [persons per kilometer squared], percentage of the population that is Black, median household income [USD], percentage of individuals renting a house or apartment, percentage of the population without a high school education, percentage of smoker rate, number of hospitals, temperature, and relative humidity), calendar year, and indicator variables for the region (Northeast, Southeast, Midwest, Southwest, and West).

^b^ The IQRs of elemental carbon, ammonium, sulfate, nitrate, soil dust, organic carbon, and PM_2.5_ mass are 0.26 μg/m^3^, 0.75 μg/m^3^, 1.73 μg/m^3^, 0.92 μg/m^3^, 0.30 μg/m^3^, 0.66 μg/m^3^, and 3.82 μg/m^3^, respectively.

^c^ Cox proportional hazard models were used to estimate the HRs.

#### Mixture Analysis Based on QGC

**Figure 1.**
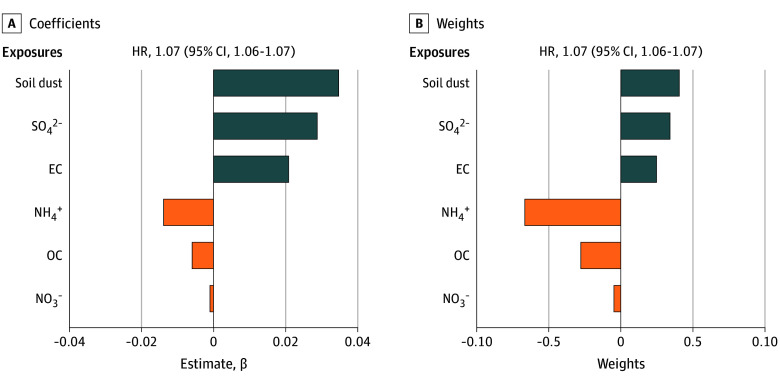
Coefficients and Weights of the Estimated Effect Size of Each Fine Particulate Matter (PM_2.5_) Major Component Associated With Depression Estimated by the Quantile G-Computation (QGC) Model The hazard ratios (HRs) and 95% CIs for per 1-quartile increase in the overall mixtures of PM_2.5_ major components are displayed above each plot and were obtained in QGC by summing the weighted averages of all coefficients. Bars representing positive effect sizes are displayed in blue and bars representing negative effect sizes are in orange. Individual coefficients (A) of each PM_2.5_ major component were calculated by multiplying weights used for each component by the sum of positive or negative coefficients. The sum of all positive weights (B) equals 1 and all negative weights equals −1. See eTable 3 in Supplement 1 for corresponding numeric data. This model was adjusted for individual-level characteristics (age, sex, race, and Medicaid eligibility), neighborhood-level socioeconomic indicators, calendar year, and indicator variables for the region. EC indicates elemental carbon; NH_4_^+^, ammonium; SO_4_^2−^, sulfate; NO_3_^−^, nitrate; OC, organic carbon.

Figure 1 and eTable 3 in Supplement 1 show the results of mixture analysis based on QGC. Overall, a 1-quartile increase in a mixture of 6 PM_2.5_ components was associated with an increased risk of depression (HR, 1.07; 95% CI, 1.06-1.07), mainly due to positive contributions from soil dust (weight = 0.41), sulfate (weight = 0.34), and elemental carbon (weight = 0.25). In addition, although ammonium, organic carbon, and nitrate had negative weights, their estimated effect size coefficients were minimal and should not be interpreted as protective.

#### Concentration Responses

**Figure 2.**
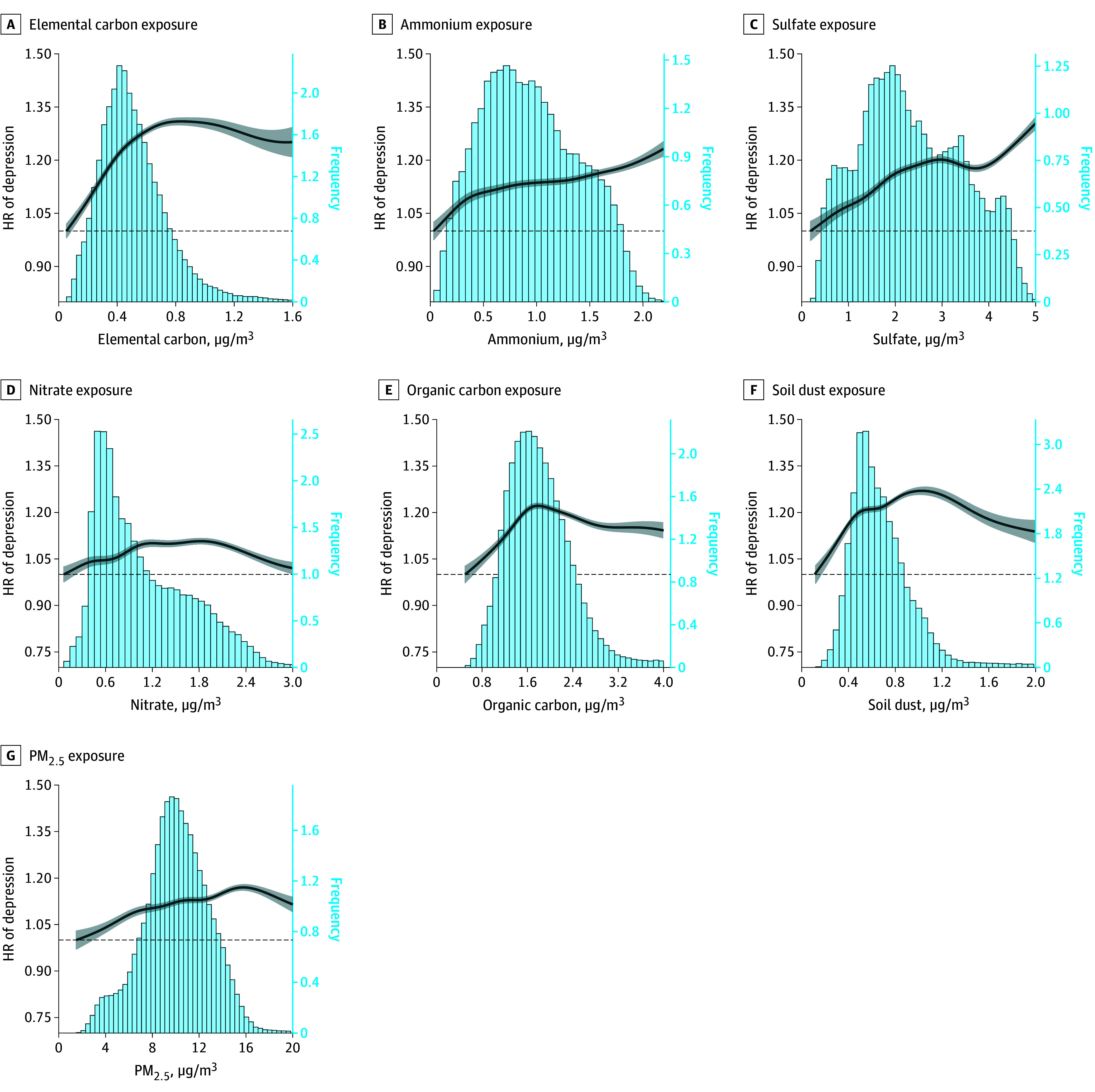
Concentration-Response Curves for Each PM_2.5_ Major Component and Depression From the Single-Pollutant Model The concentration-response curves are shown for the concentration ranges between 0.5 and 99.5 percentiles of each component. The dark blue solid lines represents the hazard ratios (HRs), and the gray shaded areas represent the 95% CIs. The dashed lines represent the reference value, where the HR is equal to 1. The bars represent histograms of distribution of PM_2.5_ and its major components. Penalized splines were applied to estimate the shape of the association of concentration with response. All models were adjusted for individual-level characteristics (age, sex, race, and Medicaid eligibility), neighborhood-level socioeconomic indicators, calendar year, and indicator variables for the region. PM_2.5_ indicates fine particulate matter.

Figure 2 displays the concentration-response curves from the single-pollutant models, showing a nearly linear increase in depression risk with PM_2.5_ and its 6 major components at low levels of exposure. The concentration response for elemental carbon and ammonium leveled off in the high range of exposure; sulfate leveled off in the medium range and still increased linearly in the high range. Nitrate, organic carbon, and soil dust appeared to have a slight linear decrease in the high exposure range.

#### Stratification Analysis

We observed heterogeneous magnitudes of associations of PM_2.5_ and its 6 major components with the risk of depression from single-pollutant models in different subpopulations with or without comorbidities (Table 3). Overall, the associations of PM_2.5_ major components, especially elemental carbon, sulfate, and soil dust with depression risk were usually more pronounced among individuals with comorbidities, such as hypertension, Alzheimer disease, dementia, chronic obstructive pulmonary disease, stroke, and congestive heart failure; there was no excess risk of depression for total PM_2.5_ among those who had no comorbidities. Additionally, we consistently found that females and Medicaid-eligible individuals (socioeconomically disadvantaged) were at a higher risk of depression associated with PM_2.5_ and its components (sulfate, ammonium, elemental carbon, and soil dust) (eTable 4 in Supplement 1).

**Table 3.** Subgroup Analysis by Comorbidities for Hazard of Depression per IQR Increase in PM_2.5_ Major Components From a Single-Pollutant Model Among the Cohort With a 5-Year Clean Period

Subgroup	Depression risk, HR (95% CI)							
	Elemental carbon	Ammonium	Sulfate	Nitrate	Soil dust	Organic carbon		PM_2.5_ mass
Any comorbidities								
No	0.91 (0.90-0.92)	0.98 (0.96-0.99)	1.03 (1.01-1.04)	0.93 (0.92-0.94)	0.95 (0.94-0.96)	1.01 (1.00-1.02)		0.98 (0.97-0.99)
Yes	1.04 (1.03-1.04)	1.01 (1.01-1.02)	1.05 (1.04-1.06)	0.99 (0.99-1.00)	1.04 (1.03-1.04)	1.00 (1.00-1.01)		1.02 (1.01-1.02)
* P* for interaction^a^	<.001	<.001	<.001	<.001	<.001	<.001		<.001
* P* value^b^	<.001	<.001	.01	<.001	<.001	.28		<.001
Alzheimer disease								
No	1.02 (1.01-1.02)	1.00 (1.00-1.01)	1.04 (1.03-1.05)	1.00 (0.98-0.99)	1.02 (1.02-1.03)	1.00 (0.99-1.00)		1.01 (1.00-1.01)
Yes	1.05 (1.04-1.06)	1.02 (1.01-1.03)	1.04 (1.03-1.05)	1.01 (1.00-1.02)	1.06 (1.05-1.07)	0.99 (0.98-1.00)		1.02 (1.01-1.02)
* P* for interaction^a^	<.001	<.001	<.001	<.001	<.001	<.001		<.001
* P* value^b^	<.001	.01	.40	<.001	<.001	.03		.03
Dementia								
No	1.01 (1.00-1.01)	1.00 (0.99-1.01)	1.04 (1.03-1.04)	0.98 (0.97-0.98)	1.01 (1.01-1.02)	1.00 (1.00-1.01)		1.01 (1.00-1.01)
Yes	1.07 (1.06-1.08)	1.04 (1.03-1.04)	1.07 (1.06-1.08)	1.02 (1.01-1.03)	1.06 (1.05-1.07)	1.00 (0.99-1.01)		1.03 (1.03-1.04)
* P* for interaction^a^	<.001	<.001	<.001	<.001	<.001	<.001		<.001
* P* value^b^	<.001	<.001	<.001	<.001	<.001	.17		<.001
Chronic obstructive pulmonary disease								
No	1.02 (1.02-1.03)	1.01 (1.01-1.02)	1.05 (1.04-1.06)	0.99 (0.99-1.00)	1.02 (1.02-1.03)	1.01 (1.00-1.01)		1.02 (1.01-1.02)
Yes	1.04 (1.04-1.05)	1.01 (1.00-1.02)	1.05 (1.04-1.06)	0.99 (0.98-1.00)	1.04 (1.04-1.05)	1.00 (0.99-1.00)		1.01 (1.01-1.02)
* P* for interaction^a^	<.001	<.001	<.001	<.001	<.001	<.001		<.001
* P* value^b^	<.001	.20	.89	.84	<.001	.01		.12
Stroke								
No	1.02 (1.02-1.03)	1.01 (1.00-1.02)	1.05 (1.04-1.05)	0.99 (0.98-0.99)	1.02 (1.02-1.03)	1.00 (1.00-1.01)		1.02 (1.01-1.02)
Yes	1.06 (1.05-1.06)	1.02 (1.01-1.03)	1.06 (1.05-1.07)	1.01 (1.00-1.01)	1.05 (1.05-1.06)	1.00 (0.99-1.00)		1.02 (1.01-1.03)
* P* for interaction^a^	<.001	<.001	<.001	<.001	<.001	<.001		<.001
* P* value^b^	<.001	.04	.08	<.001	<.001	.15		.31
Congestive heart failure								
No	1.01 (1.01-1.02)	1.01 (1.01-1.02)	1.05 (1.05-1.06)	0.99 (0.98-0.99)	1.02 (1.01-1.02)	1.01 (1.00-1.01)		1.02 (1.02-1.02)
Yes	1.05 (1.05-1.06)	1.02 (1.01-1.03)	1.06 (1.05-1.06)	1.01 (1.00-1.01)	1.05 (1.04-1.06)	0.99 (0.99-1.00)		1.02 (1.01-1.02)
* P* for interaction^a^	<.001	<.001	<.001	<.001	<.001	<.001		<.001
* P* value^b^	<.001	.39	.60	<.001	<.001	<.001		.47
Diabetes								
No	1.03 (1.02-1.03)	1.02 (1.01-1.03)	1.06 (1.05-1.06)	0.99 (0.99-1.00)	1.03 (1.02-1.03)	1.01 (1.00-1.01)		1.02 (1.02-1.03)
Yes	1.03 (1.03-1.04)	1.01 (1.00-1.02)	1.04 (1.04-1.05)	0.99 (0.98-1.00)	1.04 (1.03-1.05)	0.99 (0.99-1.00)		1.01 (1.01-1.02)
* P* for interaction^a^	<.001	<.001	<.001	<.001	<.001	<.001		<.001
* P* value^b^	.21	.04	.03	.35	.01	.001		.03
Hypertension								
No	0.97 (0.96-0.97)	1.01 (1.00-1.02)	1.06 (1.05-1.07)	0.97 (0.96-0.98)	0.98 (0.97-0.99)	1.01 (1.00-1.02)		1.01 (1.00-1.02)
Yes	1.04 (1.03-1.04)	1.01 (1.01-1.02)	1.05 (1.05-1.06)	0.99 (0.99-1.00)	1.04 (1.03-1.04)	1.00 (1.00-1.01)		1.02 (1.01-1.02)
* P* for interaction^a^	<.001	<.001	<.001	<.001	.01	<.001		<.001
* P* value^b^	<.001	.57	.14	<.001	<.001	.06		.13
Lung cancer								
No	1.03 (1.03-1.04)	1.01 (1.01-1.020)	1.05 (1.04-1.06)	0.99 (0.99-1.00)	1.03 (1.03-1.04)	1.00 (1.00-1.01)		1.02 (1.01-1.02)
Yes	1.05 (1.04-1.06)	1.00 (0.99-1.02)	1.05 (1.03-1.07)	1.00 (0.99-1.02)	1.04 (1.03-1.05)	1.00 (0.99-1.01)		1.01 (1.00-1.02)
* P* for interaction^a^	<.001	<.001	<.001	<.001	<.001	<.001		<.001
* P* value^b^	.01	.22	.93	.25	.49	.26		.46

Abbreviations: HR, hazard ratio; PM_2.5_, fine particulate matter.

^a^ *P* for interaction was assessed by including interaction terms between these potential modifiers and air pollutants.

^b^ *P* value for interaction (Wald test comparing subgroup coefficients) was reported as a complementary measure to summarize heterogeneity across comorbidity groups.

### Sensitivity Analysis

Our results were robust across multiple sensitivity analyses. First, multipollutant Cox models showed consistent positive associations for elemental carbon, sulfate, ammonium, and soil dust (eTable 5 in Supplement 1), aligning with findings from QGC (eTable 6 in Supplement 1). Second, the single-pollutant Cox model and the QGC yielded results after accounting for residual PM_2.5_ (eTable 6 in Supplement 1). Third, using an alternative exposure data set confirmed consistency (eTable 7 in Supplement 1). Fourth, applying both a less strict 3-year and a stricter 10-year clean period yielded similar associations (eTable 8 in Supplement 1). Fifth, varying exposure lags (0, 1, and 1-3 years) showed stable positive associations for elemental carbon, sulfate, soil dust, ammonium, and PM_2.5_ mass (eTable 9 in Supplement 1). Finally, analyses addressing outcome misclassification—using linear regression and correction for Medicare’s sensitivity and specificity—suggested a slight bias toward the null (eTable 10 and eTable 11 in Supplement 1).

## Discussion

In this large national longitudinal cohort study, we found that long-term exposure to PM_2.5_ mass and 4 of its major components (sulfate, soil dust, ammonium, and elemental carbon) was associated with a higher risk of depression. QGC confirmed that the combined positive association of PM_2.5_ mixtures with depression risk was much higher than PM_2.5_ alone, and further revealed that soil dust, sulfate, and elemental carbon were the most responsible for the observed associations. Although the individual-level effect sizes were modest, such small relative increases in risk may have meaningful population-level implications given the widespread exposure to air pollution and the high burden of depression among older adults. The subgroup results consistently showed that those observed associations were more pronounced among older adults who experienced various comorbidities, particularly those with cardiometabolic and neurologic diseases.

Our findings substantiate the existing evidence on the adverse associations of PM_2.5_ with depression risk. Although increasing population studies have shown that exposure to PM_2.5_ was associated with a higher risk of depression,^6,7,8,9,10^ current literature on exposure to PM_2.5_ components and depression remains limited.^18,20,21^ Consistent with our findings in the single-pollutant model, a prospective cohort study conducted among 9503 Chinese adults indicated significant positive associations of long-term exposure to black carbon, ammonium, and sulfate with the worsening of depressive symptoms.^20^ Similarly, Liu et al^21^ reported a significant positive association of long-term exposure to black carbon and sulfate with depression prevalence among 5707 middle-aged and older adults in China. However, those studies examined PM_2.5_ components individually, which may not adequately capture the combined associations of PM_2.5_ components with depression due to the potential synergistic interactions between PM_2.5_ components.^34,35^ Utilizing QGC, a mixture approach that considers all components simultaneously proves to be a more comprehensive approach to assess the overall associations of PM_2.5_ components and identify the most significant contributors. To our knowledge, only 1 study^24^ has used the QGC model to explore the association of long-term exposure to a mixture of 5 major PM_2.5_ components (elemental carbon, sulfate, nitrate, organic carbon, and ammonium) with depressive symptoms, which was a follow-up study of a Chinese cohort. While there is a discrepancy between our findings from the US Medicare data on clinical depression and their findings on depressive symptoms regarding nitrate, both studies observed similar patterns for other components. Specifically, exposure to the mixture—primarily due to sulfate and elemental carbon—was significantly positively associated with depression, while nitrate and organic carbon exhibited negative associations. Notably, the exposure-response curves for several components (eg, organic carbon, nitrate, and soil dust) showed a slight downturn at higher concentrations. This apparent attenuation likely reflects sparse data and greater uncertainty in the upper exposure range, as well as potential competing risks, or depletion of susceptible individuals.^36,37^

Sulfate, a secondary inorganic aerosol mainly from fossil fuel combustion, commonly exists as (NH_4_)_2_SO_4_, NH_4_HSO_4_, or sulfuric acid. It may contribute to depression by inducing mitochondrial dysfunction^38^ and oxidative stress^39,40^ that affect the central nervous system. Additionally, sulfate may act as a proxy for other combustion-related pollutants or increase the solubility and bioavailability of trace metals due to aerosol acidity.^41^ Elemental carbon, primarily emitted from traffic and biomass burning,^42,43^ may exert neurotoxic effects through oxidative stress and systemic inflammation.^44^ Elemental carbon particles from traffic sources are small (50 nm) and can easily bypass the blood-brain barrier, leading to neurotoxicity.^45^ Moreover, elemental carbon can adsorb polycyclic aromatic hydrocarbons^46^ and metals,^47^ potentially leading to neurotoxic effects upon combination. Soil dust, rich in silica and metals like iron and calcium, may originate from both natural and anthropogenic sources. The neurotoxicity of soil dust may be attributed to its heavy metal content because these metals can accumulate in the blood and bones, causing damage to the nervous system.^48,49^ Silica has been shown to induce oxidative stress and systemic inflammation,^50,51^ both of which are associated with an increased risk of depression.^52^ While nitrate and organic carbon showed smaller or inverse associations in the QGC, their associations were notably lower compared with the positive associations of soil dust, elemental carbon, and sulfate. Our study emphasizes the importance of performing a mixture analysis for exposure to multiple pollutants.

Interestingly, we found that those individuals with various comorbidities, particularly hypertension, cardiovascular diseases (eg, stroke and congestive heart failure), neurodegenerative diseases (eg, Alzheimer disease and dementia), and respiratory diseases (eg, chronic obstructive pulmonary disease), were at a significantly higher risk of depression when exposed to total PM_2.5_ mass and its major components. Our finding suggests that comorbidities might interact synergistically with PM_2.5_ mass and its component exposure to further accelerate the development of depression. Consistent with our findings, a population-based longitudinal cohort study using the Medicare data from 2000 to 2016 also reported that the positive associations of long-term exposure to PM_2.5_ with depression risk were only observed and more evident among individuals with comorbidities such as hypertension, Alzheimer disease, dementia, cancers, or stroke.^9^ The potential explanation is that older adults with comorbidities were more likely to have a higher chance of exposure to complex medication, weaker social engagement, and unhealthy lifestyle, all of which make individuals’ nervous systems more vulnerable to certain preinflammatory factors that are susceptible to neuroinflammatory triggers (eg, air pollution).^53^ We also observed that the associations of certain PM_2.5_ components, such as sulfate and ammonium, with depression were less pronounced in individuals with metabolic diseases like diabetes compared with those without diabetes. This finding may be attributed to the use of anti-inflammatory medications or lifestyle modifications that help reduce systemic inflammation triggered by air pollutants. However, these findings should be interpreted cautiously given the relatively small sample size of individuals without comorbidities.

### Strengths and Limitations

This study has several notable strengths. First, to our knowledge, it is the first nationwide, population-based, large-scale open cohort study conducted in the US to investigate the joint association of 6 major PM_2.5_ components with the onset of depression in elderly individuals. Our use of QGC effectively managed collinear exposures and assessed the contribution of each PM_2.5_ component in the mixture. Second, we applied more comprehensive Medicare claims, including doctor visits, to capture depression cases which might not result in hospitalization. Third, the requirements of a 5-year clean period enabled us to yield more accurate results for depression incidence rather than prevalence, preferable to assessing important risk factors.

Despite these strengths, some limitations of this study should also be noted. First, measurement error of exposure may exist because we were unable to obtain the participants’ residential address information and thus evaluate the exposure based on the zip code level instead of individual level. Second, while 6 PM_2.5_ major components examined in our study accounted for most of total PM_2.5_ mass, other PM_2.5_ components such as trace elements were not considered in our analysis. Third, although QGC can accommodate correlated exposures, it cannot fully address multicollinearity among highly correlated PM_2.5_ components when estimating the effect sizes of individual components, such as ammonium and sulfate. Fourth, although our models adjusted for various covariates, we cannot completely exclude the possibility that unmeasured individual-level risk factors (eg, physical exercise, smoking, and alcohol consumption) or residual confounding may have biased the observed risk estimates. Fifth, the validity of a diagnosis of depression in the Medicare database has not been thoroughly investigated, and outcome misclassification is a concern when relying on administrative records. However, because misclassification is likely nondifferential (unrelated to exposure), our sensitivity analyses also suggest that this would likely bias the results toward the null.

## Conclusions

The results of this US cohort study among older adults found that long-term exposure to PM_2.5_ and its major components, notably from soil dust, sulfate and elemental carbon, was associated with a greater risk of depression, especially among individuals who experienced various comorbidities. Our findings underscore the necessity for stricter regulation of ambient PM_2.5_ emissions, particularly from fossil fuel combustion and traffic sources.

## Notes

Supplement 1. eMethods. eTable 1. ICD-codes for depression condition used in Medicare CCW database eFigure 1. Nationwide occurrences of first depression events per 100,000 Medicare beneficiaries across the contiguous United States (2000 to 2018), with a 5-y clean period considered eTable 2. The distribution of annual PM2.5 total mass and its major component (µg/m3) concentrations eFigure 2. The Pearson correlation matrix among PM2.5 mass and PM2.5 major components (µg/m3) eFigure 3. Pie chart of the mean chemical composition of PM2.5 mass concentrations eFigure 4. Average concentrations of PM2.5 major components (µg/m3) across the contiguous United States from 2000 to 2018 eTable 3. Associations between PM2.5 major components and depression estimated by quantile g-computation model among the cohort with 5-year clean period eTable 4. Subgroup analysis by characteristics of hazard ratios and 95% CIs of per IQR increase in PM2.5 major components associated with depression from single-pollutant models among the cohort with 5-year clean period eTable 5. Hazard ratios and 95% CIs of per IQR increase in PM2.5 major components associated with depression from single-pollutant models and multi-pollutant models, among the cohort with 5-year clean period eTable 6. Associations between PM2.5 major components and depression estimated by quantile g-computation model among the cohort with 5-year clean period in the main and sensitivity analyses eTable 7. Hazard ratios and 95% CIs of per IQR increase in PM2.5 major components associated with depression using different exposure data from single-pollutant models among the cohort with 5-year clean period eTable 8. Hazard ratios and 95% CIs of per IQR increase in PM2.5 major components associated with depression using varying clean periods from single-pollutant model eTable 9. Hazard ratios and 95% CIs of per IQR increase in PM2.5 major components associated with depression using varying lag periods from single-pollutant models among the cohort with 5-year clean period eTable 10. Hazard ratios and 95% CIs of per IQR increase in PM2.5 major components associated with depression from single-pollutant models among the cohort with 5-year clean period, derived from the linear rate models eTable 11. Hazard ratios and 95% CIs of per IQR increase in PM2.5 major components associated with depression from single-pollutant models among the cohort with 5-year clean period, accounting for potential outcome misclassification via adjusting data for assumed sensitivity and specificity of classification eReferences.

Supplement 2. Data Sharing Statement
