# Supplementary material for: Exposure to Multiple Fine Particulate Matter Components and Incident Depression in the US Medicare Population
Source: JAMA Netw Open. 2025 Dec 22;8(12):e2551042. doi: 10.1001/jamanetworkopen.2025.51042 (PMC12723547; doi:10.1001/jamanetworkopen.2025.51042)
Supplement: Supplement 1. — eMethods. eTable 1. ICD-codes for depression condition used in Medicare CCW database eFigure 1. Nationwide occurrences of first depression events per 100,000 Medicare beneficiaries across the contiguous United States (2000 to 2018), with a 5-y clean period considered eTable 2. The distribution of annual PM2.5 total mass and its major component (µg/m3) concentrations eFigure 2. The Pearson correlation matrix among PM2.5 mass and PM2.5 major components (µg/m3) eFigure 3. Pie chart of the mean chemical composition of PM2.5 mass concentrations eFigure 4. Average concentrations of PM2.5 major components (µg/m3) across the contiguous United States from 2000 to 2018 eTable 3. Associations between PM2.5 major components and depression estimated by quantile g-computation model among the cohort with 5-year clean period eTable 4. Subgroup analysis by characteristics of hazard ratios and 95% CIs of per IQR increase in PM2.5 major components associated with depression from single-pollutant models among the cohort with 5-year clean period eTable 5. Hazard ratios and 95% CIs of per IQR increase in PM2.5 major components associated with depression from single-pollutant models and multi-pollutant models, among the cohort with 5-year clean period eTable 6. Associations between PM2.5 major components and depression estimated by quantile g-computation model among the cohort with 5-year clean period in the main and sensitivity analyses eTable 7. Hazard ratios and 95% CIs of per IQR increase in PM2.5 major components associated with depression using different exposure data from single-pollutant models among the cohort with 5-year clean period eTable 8. Hazard ratios and 95% CIs of per IQR increase in PM2.5 major components associated with depression using varying clean periods from single-pollutant model eTable 9. Hazard ratios and 95% CIs of per IQR increase in PM2.5 major components associated with depression using varying lag periods from single-pollutant models among the cohort with [file jamanetwopen-e2551042-s001.pdf]

Supplemental Online Content

Deng Y, Hao H Zhu Q, Liu Y, Steenland K. Exposure to multiple $PM_{2.5}$ Major components and incident depression in the US Medicare population. *JAMA Netw. Open*. 2025;8(12):e2551042. doi:10.1001/jamanetworkopen.2025.51042

eMethods.
eTable 1. *ICD*-codes for depression condition used in Medicare CCW database
eFigure 1. Nationwide occurrences of first depression events per 100,000 Medicare beneficiaries across the contiguous United States (2000 to 2018), with a 5-y clean period considered
eTable 2. The distribution of annual PM2.5 total mass and its major component (μg/m3) concentrations
eFigure 2. The Pearson correlation matrix among PM2.5 mass and PM2.5 major components (μg/m3)
eFigure 3. Pie chart of the mean chemical composition of PM2.5 mass concentrations
eFigure 4. Average concentrations of PM2.5 major components (μg/m3) across the contiguous United States from 2000 to 2018
eTable 3. Associations between PM2.5 major components and depression estimated by quantile g-computation model among the cohort with 5-year clean period
eTable 4. Subgroup analysis by characteristics of hazard ratios and 95% CIs of per IQR increase in PM2.5 major components associated with depression from single-pollutant models among the cohort with 5-year clean period
eTable 5. Hazard ratios and 95% CIs of per IQR increase in PM2.5 major components associated with depression from single-pollutant models and multi-pollutant models, among the cohort with 5-year clean period
eTable 6. Associations between PM2.5 major components and depression estimated by quantile g-computation model among the cohort with 5-year clean period in the main and sensitivity analyses
eTable 7. Hazard ratios and 95% CIs of per IQR increase in PM2.5 major components associated with depression using different exposure data from single-pollutant models among the cohort with 5-year clean period
eTable 8. Hazard ratios and 95% CIs of per IQR increase in PM2.5 major components associated with depression using varying clean periods from single-pollutant model
eTable 9. Hazard ratios and 95% CIs of per IQR increase in PM2.5 major components associated with depression using varying lag periods from single-pollutant models among the cohort with 5-year clean period
eTable 10. Hazard ratios and 95% CIs of per IQR increase in PM2.5 major components associated with depression from single-pollutant models among the cohort with 5-year clean period, derived from the linear rate models
eTable 11. Hazard ratios and 95% CIs of per IQR increase in PM2.5 major components associated with depression from single-pollutant models among the cohort with 5-year clean period, accounting for potential outcome misclassification via adjusting data for assumed sensitivity and specificity of classification
eReferences

This supplemental material has been provided by the authors to give readers additional information about their work.

eMethods

Exposure assessment

The training data of $PM_{2.5}$ major components were collected from 987 monitoring sites, and hundreds of additional predictors (e.g., satellite observations, traffic counts, and meteorological variables) were applied for multiple superlearning models and an ensemble weighted-averaging model. The concentrations of total $PM_{2.5}$ mass were estimated by an ensemble model that included hundreds of predictors and several machine learners.[1] We finally averaged these gridded predictions for total $PM_{2.5}$ mass and its major components at the ZIP code level for each year and assigned concentrations of these exposures to each Medicare beneficiary based on their residential ZIP code and the calendar year. Because residential ZIP codes in the Medicare data are updated annually, our exposure assessment accounted for participants' residential moves over time. Time-varying 5-year moving averages of exposure preceding each follow-up year were calculated for each Medicare beneficiary.

Outcome assessment

We established a 5-year clean period after enrollment, during which no depression-related diagnosis codes were recorded. This approach aimed to exclude potentially prevalent cases during the initial years of follow-up, making it more likely that any diagnosis made afterward reflected disease incidence. While we are analyzing the first occurrence of depression among our subjects, we believe that using a 5-year clean period for the first occurrence in Medicare data can approximate true incidence. This approach reduces the likelihood that subjects had been diagnosed with depression prior to entering our cohort. We considered 5 years a reasonably sufficient duration to confirm that individuals were not diagnosed with depression prior to their Medicare diagnosis according to a previous study.[2] In the final cohort, study participants were followed from January 1 of the first year after the clean period until the

occurrence of a depression diagnosis, death, or the end of the follow-up period. Follow-up began at the start of the first eligible year after the 5-year clean period, and person-time before cohort entry was not included to avoid immortal time bias.

Covariates

First, we gathered four individual-level demographic characteristics from the Medicare denominator file: age at entry, sex, Medicaid eligibility (categorized as eligible or not, with eligibility serving as a proxy for low socioeconomic status),[3] and self-reported race (categorized as White, Black, and other, which encompasses Hispanic, Asian, Alaskan Native or American Indian, and unknown).[3-4] The racial categorization was designed to ensure stable statistical estimation, as further subdivisions were not feasible due to the limitations of the data structure provided by CMS. Additionally, we collected 10 neighborhood-level socioeconomic indicators, including six ZIP code-level variables (percentage below the poverty line, population density, median household income, percentage of Black individuals, percentage of the population renting a house or apartment, and percentage of the population who have not graduated from high school), county-level behavioral risk factors (smoking rate), healthcare capacity variables (number of hospitals), and area-level annual average meteorology (temperature and relative humidity). To account for potential residual confounding from spatial and temporal trends, we also incorporated region (Midwest, Southwest, Southeast, Northeast, and West) and calendar year indicators. Six ZIP code-level variables were sourced from the American Community Survey for 2005–2019,[5] as well as the 2000 and 2010 U.S. Census data.[6-7] County-level behavioral risk factors were obtained from the Behavioral Risk Factor Surveillance System (BRFSS) spanning 2000 to 2016.[8] Healthcare capacity data were sourced from the 2010, 2015, and 2018 American Hospital Association Annual Survey Database.[9] Missing data were extremely rare, representing <0.1% of our data. Any missing data were

© 2025 Deng Y et al. *JAMA Network Open.*

linearly interpolated or extrapolated based on the data available to maintain consistency with the methodology used in the Medicare population of previous studies.[10-12]

Statistical analysis.

Single-pollutant Cox proportional hazard models were applied to estimate hazard ratios (HRs) and 95% confidence intervals (CIs) for per interquartile range (IQR) increase in the 5-year average exposure of each $PM_{2.5}$ component, considering a 5-year clean period. All models were stratified by Medicaid eligibility (eligible or not for Medicaid), age at entry (1-y age categories), sex (male vs. female), and race (White, Black, and other), as well as adjusted for the neighborhood-level covariates (see Covariates).

Quantile g-computation uses a Cox proportional hazard model-based approach combined with g-computation to assess the effects of a simultaneous one-quartile increase in all exposures within the mixture. Each component is assigned a positive or negative weight according to the direction of its independent effect. The specific weights for the individual mixture components sum to 1 in either the positive or negative direction and cannot be directly compared. In order to directly compare the effect of each pollutant on outcomes, we calculate the individual coefficients of each component by multiplying their weights by the sum of the positive or negative coefficients. We included six major $PM_{2.5}$ components simultaneously into one model using quantile g-computation. The same covariates as in the original Cox model were used in quantile g-computation.

We conducted several stratification analyses by demographics [sex (male vs. female), race (white, black, and other), Medicaid eligibility (ineligible vs. ever eligible), age (≤75 years vs. >75 years), region (Midwest, Southwest, Southeast, Northeast, and West)] and various comorbidities available from the Medicare data to determine which subpopulations may be more susceptible than others. P-values for interaction were assessed by including interaction terms between these potential modifiers and air pollutants, as well as by the Wald

© 2025 Deng Y et al. *JAMA Network Open.*

test.[13] Comorbid conditions were identified based on the first recorded diagnosis using an algorithm that incorporated Medicare claims data with valid ICD-9 or ICD-10 codes.[14] These conditions included Alzheimer's disease (AD, ICD-9 code 331; ICD-10 code G30), dementia (ICD-9 code 290, 294, 331; ICD-10 codes F01–F06, G30–G31), chronic obstructive pulmonary disease (COPD, ICD-9 codes 490–492, 494, 496; ICD-10 codes J40–44, J47), hypertension (ICD-9 codes 401–405; ICD-10 codes I10–I13, I15), diabetes (ICD-9 codes 249–250; ICD-10 codes E08–E11, E13), congestive heart failure (CHF, ICD-9 codes 402, 404, 428; ICD-10 codes I11, I13, I50), stroke (ICD-9 codes 430, 431, 433–436; ICD-10 codes G45–46, I60, I61, I63–I67), and lung cancer (ICD-9 code 162; ICD-10 codes C34). The comorbidities occurred before or in the same year as the depression diagnosis, indicating that the comorbidities likely developed before depression. For instance, the 'no dementia' group was composed of those who never had dementia prior to their first diagnosis of depression.

We performed several sensitivity analyses to evaluate the robustness of our main findings. First, considering the high correlations between SO_4^{2-} and NH_4^+ (Pearson correlation coefficients = 0.84, eFigure 2 in Supplement 1), we fitted two separate multi-pollutant models based on the Cox model, 1) by including EC, SO_4^{2-}, NO_3^-, OC, and DUST simultaneously and 2) by including EC, NH_4^+, NO_3^-, OC, and DUST simultaneously in one model. Additionally, we employed quantile g-computation to fit two separate models to maintain consistency with the Cox model and compared the results between quantile g-computation and Cox model. Second, to account for the influence of other $PM_{2.5}$ components, we further adjusted for the residual $PM_{2.5}$ mass in the single-pollutant models, which was derived by subtracting the component of interest from the total $PM_{2.5}$ mass. Quantile g-computation also evaluated the joint effect of the six primary $PM_{2.5}$ components alongside other components (total $PM_{2.5}$ minus the sum of the six key components) in the mixture.

© 2025 Deng Y et al. *JAMA Network Open.*

Third, we assessed the association between depression and $PM_{2.5}$ and its major components in the single-pollutant model by using an alternative validated exposure dataset reported by van Donkelaar et al.'s[15] to evaluate whether the results were consistent with different exposure data. Fourth, we applied both a less strict 3-year and a stricter 10-year "clean period" prior to the depression diagnosis to evaluate whether varying clean periods influenced the observed associations. Fifth, we assessed different lag years for exposure (0-year lag, 1-year lag, and average exposure of 1-3 years lag) to determine whether exposure accelerates depression progression or influences its onset. We hypothesize that a shorter lag suggests $PM_{2.5}$ accelerates existing depression, while a longer lag may reflect its effect on the earlier stages, such as the onset of depression. Finally, we evaluated the potential effect of outcome misclassification using two approaches: (1) we applied linear regression models with GEE to analyze depression incidence rates. This method offered additive effect estimates that were less susceptible to bias, since random misclassification of outcomes was integrated into the residual errors, reflecting true event rates;[16] (2) we accounted for the effects of misclassification by following methods comparable to those outlined by Fox et al.[17] and adjusting the observed outcomes in each stratum with estimates of Medicare specificity and sensitivity from Hwang et al.[18] to better approximate the true case numbers.

© 2025 Deng Y et al. *JAMA Network Open.*

eTable 1. ICD-codes for depression condition used in Medicare CCW database.

	Depression
ICD-9[1]	296.20, 296.21, 296.22, 296.23, 296.24, 296.25, 296.26, 296.30, 296.31, 296.32, 296.33, 296.34, 296.35, 296.36, 296.51, 296.52, 296.53, 296.54, 296.55, 296.56, 296.60, 296.61, 296.62, 296.63, 296.64, 296.65, 296.66, 296.89, 298.0, 300.4, 309.1, 311 (any DX on the claim)
ICD-10[a]	F31.30, F31.31, F31.32, F31.4, F31.5, F31.60, F31.61, F31.62, F31.63, F31.64, F31.75, F31.76, F31.77, F31.78, F31.81, F32.0, F32.1, F32.2, F32.3, F32.4, F32.5, F32.9, F33.0, F33.1, F33.2, F33.3, F33.40, F33.41, F33.42, F33.8, F33.9, F34.1, F43.21, F43.23 (any DX on the claim)
Number/Type of Claims to Qualify[b]	At least 1 inpatient, SNF, HHA, HOP, or Carrier claim with DX codes

Abbreviations: ICD, International Classification of Diseases; CCW, Chronic Conditions Warehouse.

[a]ICD-10 codes are effective 10/2015; effective dates for ICD-9 codes vary, but are valid through 09/2015. Researchers may be interested in confirming the code(s) of interest in the accompanying claims data files.

[b]SNF refers to skilled nursing facility; HHA refers to home health agency; HOP refers to hospital outpatient. Carrier claims refer to claim types 71 and 72 (not durable medical equipment [DME] claim types 81 or 82), and excludes any claims for which line item Berenson-Eggers Type of Service (BETOS) code variable equals D1A, D1B, D1C, D1D, D1E, D1F, D1G (which is DME), or O1A (which is ambulance services). The intent of the algorithm is to exclude claims where the services do not require a licensed health care professional. When two claims are required, they must occur at least one day apart. DX denotes diagnosis.

© 2025 Deng Y et al. *JAMA Network Open.*

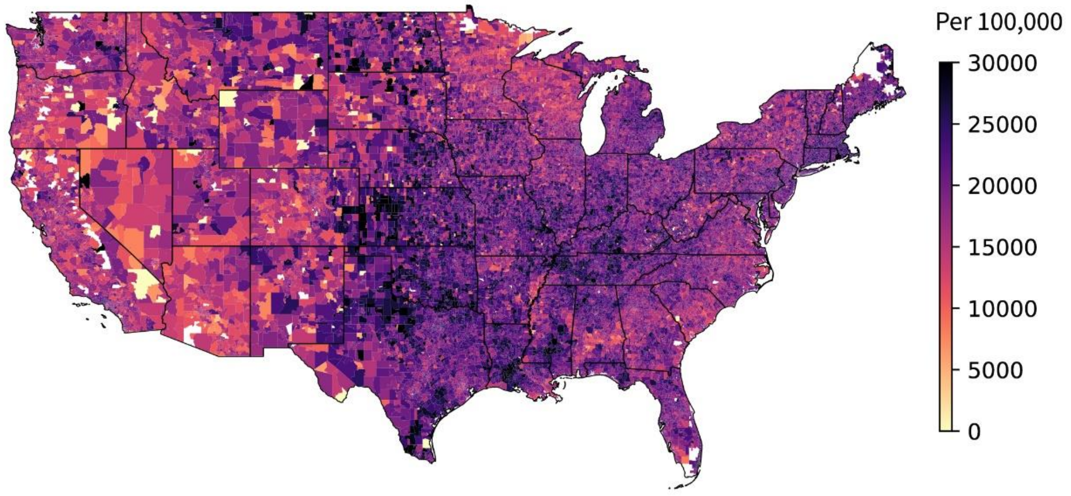

eFigure 1. Nationwide occurrences of first depression events per 100,000 Medicare beneficiaries across the contiguous United States (2000 to 2018), with a 5-y clean period considered.

© 2025 Deng Y et al. *JAMA Network Open*.

eTable 2. The distribution of annual $PM_{2.5}$ total mass and its major component (μg/m^3) concentrations.

Exposure	Min	Percentile					Max	Mean (SD)
		5th	25th	50th	75th	95th		
Elemental carbon	0.05	0.25	0.40	0.52	0.66	0.93	2.12	0.55 (0.22)
Ammonium	0.03	0.29	0.61	0.95	1.36	1.74	2.42	0.98 (0.46)
Sulfate	0.19	0.72	1.61	2.39	3.33	4.32	5.84	2.46 (1.10)
Nitrate	0.06	0.42	0.63	0.97	1.56	2.28	4.78	1.13 (0.61)
Soil dust[a]	0.12	0.35	0.49	0.60	0.78	1.11	3.14	0.66 (0.27)
Organic carbon	0.50	1.12	1.47	1.76	2.14	2.79	5.48	1.84 (0.54)
$PM_{2.5}$ mass	0.63	5.25	8.35	10.17	12.16	14.72	26.83	10.19 (2.86)

Note: $PM_{2.5}$, fine particulate matter; SD, standard deviation.

[a]Soil dust was calculated using an empirical formula derived from a previous study,[19] which is (2.20×Al + 2.49×Si + 1.63×Ca + 1.94×Ti + 2.42×Fe). However, due to the absence of Aluminum (Al) and Titanium (Ti) data, we adapted the formula to (2.49×Si + 1.63×Ca + 2.42×Fe)×1.4 to compensate for the concentration levels. The factor of 1.4 is the adjusted slope for DUST based on the findings in van Donkelaar et al.[15]

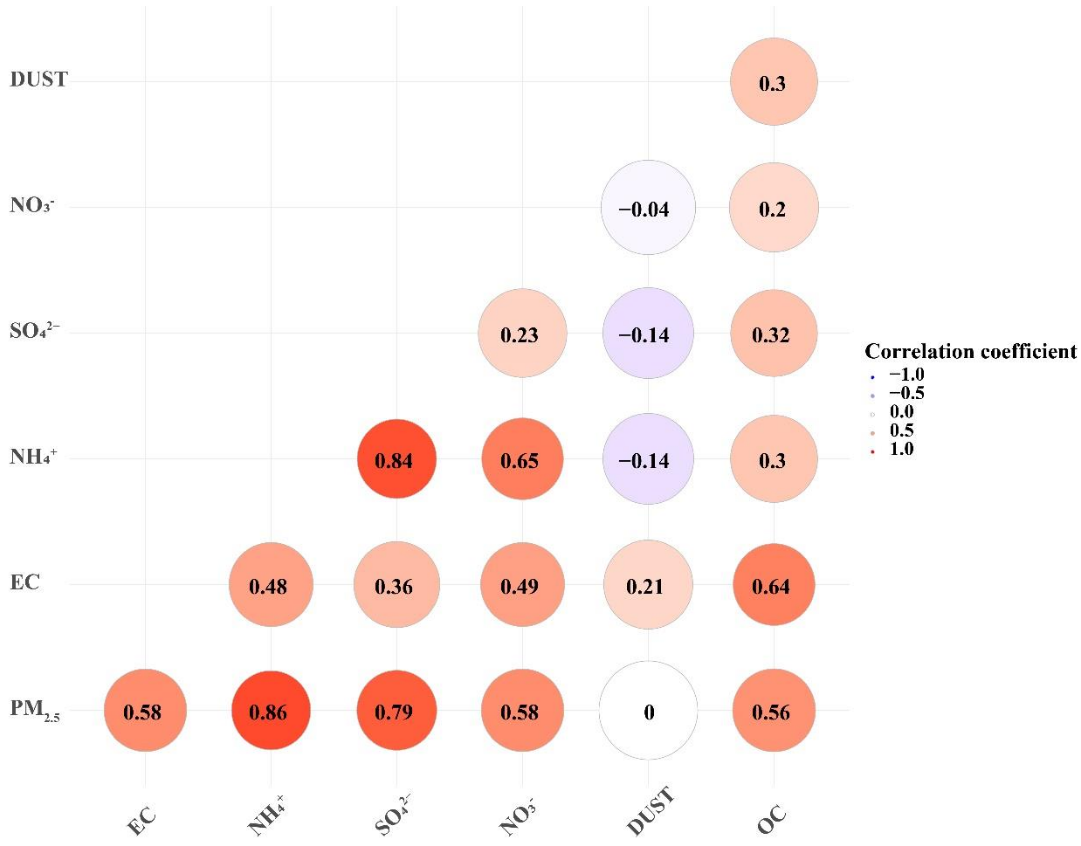

eFigure 2. The Pearson correlation matrix among $PM_{2.5}$ mass and $PM_{2.5}$ major components.

Abbreviations: EC, elemental carbon; NH_4^+, ammonium; SO_4^{2-}, sulfate; NO^{3-}, nitrate; DUST, soil dust; OC, organic carbon; $PM_{2.5}$, fine particulate matter.

© 2025 Deng Y et al. *JAMA Network Open*.

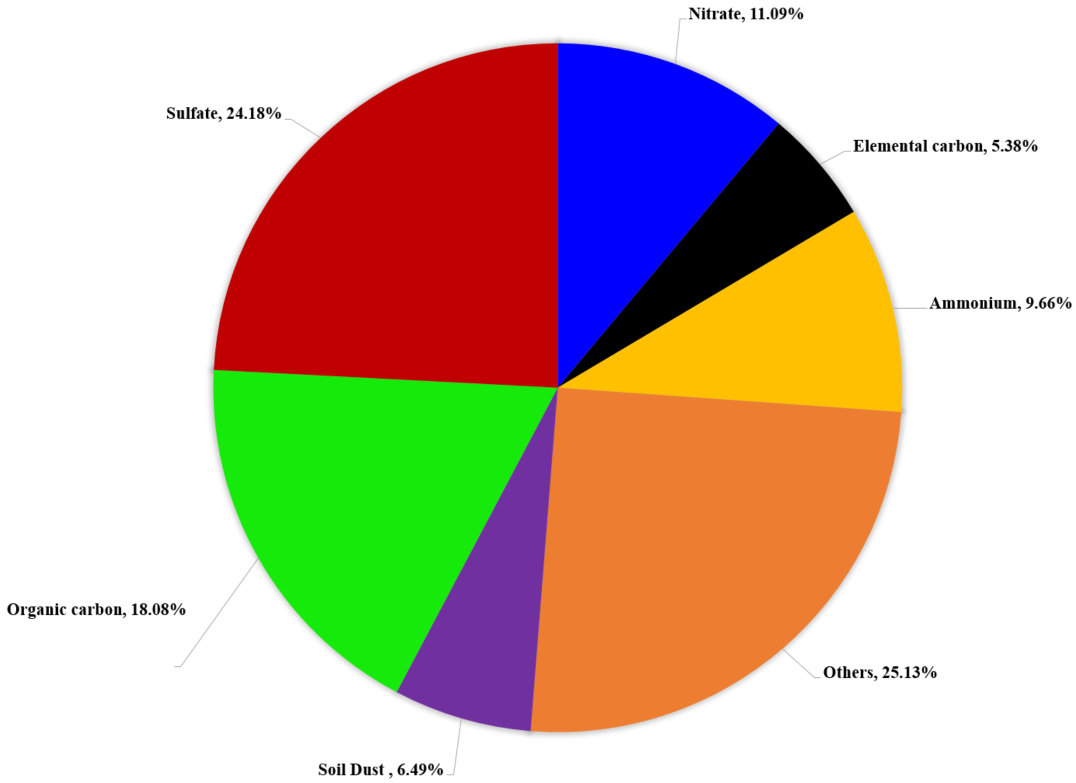

eFigure 3. Pie chart of the mean chemical composition of $PM_{2.5}$ mass concentrations.

© 2025 Deng Y et al. *JAMA Network Open*.

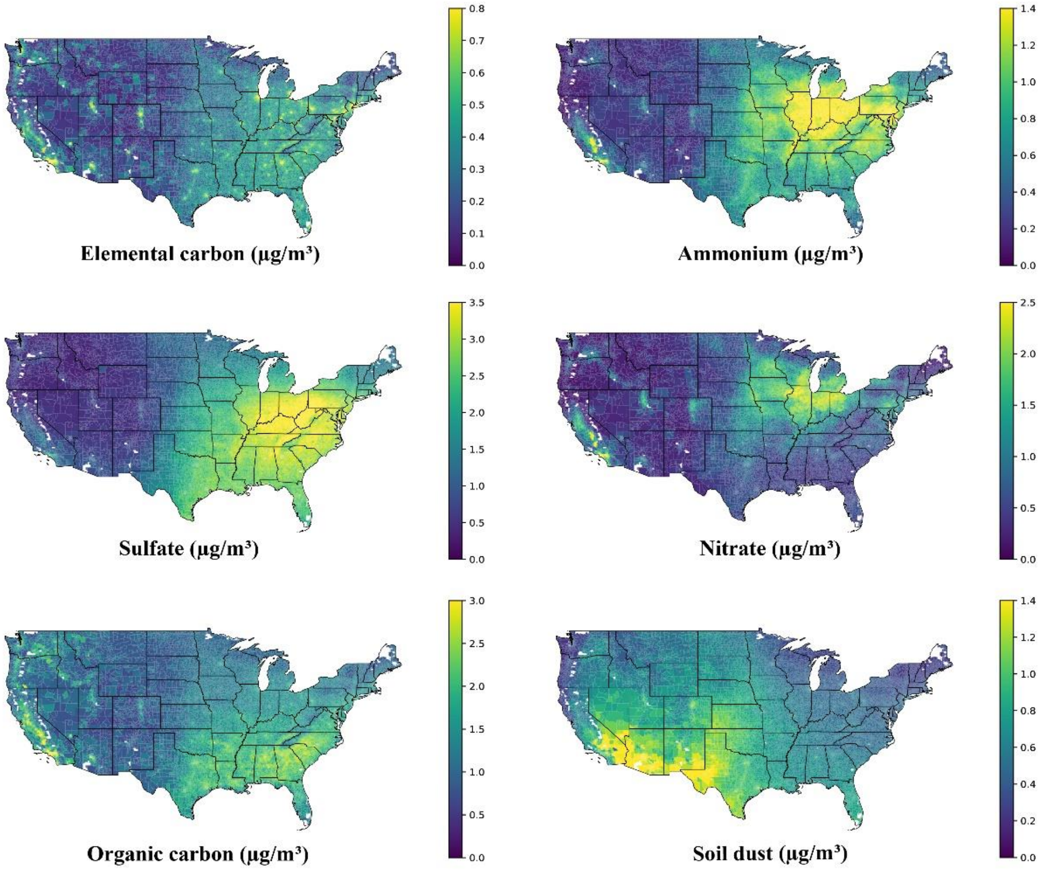

eFigure 4. Average concentrations of $PM_{2.5}$ major components (μg/m^3) across the contiguous United States from 2000 to 2018.

© 2025 Deng Y et al. *JAMA Network Open*.

eTable 3. Associations between $PM_{2.5}$ major components and depression estimated by quantile g-computation model among the cohort with 5-year clean period.

Air pollutants	Weights	Estimate (β)	HR (95% CI)
$PM_{2.5}$ mixtures		0.06	1.07 (1.06, 1.07)
Elemental carbon	0.25	0.02	
Ammonium	-0.67	-0.01	
Sulfate	0.34	0.03	
Nitrate	-0.05	-0.001	
Soil dust	0.41	0.04	
Organic carbon	-0.28	-0.01	

Abbreviations: CI, confidence intervals; HR, Hazard ratios; $PM_{2.5}$, fine particulate matter.
Table for the numeric data [weights, estimate, HR (95% CI)] corresponding to Figure 1.

© 2025 Deng Y et al. *JAMA Network Open.*

eTable 4. Subgroup analysis by characteristics of hazard ratios and 95% CIs of per IQR increase in $PM_{2.5}$ major components associated with depression from single-pollutant models among the cohort with 5-year clean period.

Subgroup	Elemental carbon	Ammonium	Sulfate	Nitrate	Soil dust	Organic carbon	$PM_{2.5}$ mass
Age at entry (years)							
<75	1.03 (1.02, 1.03)	1.02 (1.01, 1.03)	1.07 (1.06, 1.07)	1.00 (0.99, 1.00)	1.02 (1.01, 1.02)	1.02 (1.01, 1.02)	1.03 (1.02, 1.03)
≥75	1.04 (1.03, 1.04)	1.00 (1.00, 1.01)	1.04 (1.03, 1.05)	0.99 (0.99, 1.00)	1.05 (1.04, 1.06)	0.99 (0.98, 0.99)	1.01 (1.00, 1.01)
P for interaction	<0.001	<0.001	<0.001	<0.001	<0.001	<0.001	<0.001
Sex							
Male	1.02 (1.02, 1.03)	1.01 (1.00, 1.01)	1.04 (1.03, 1.05)	0.99 (0.99, 1.00)	1.03 (1.03, 1.04)	0.99 (0.99, 1.00)	1.01 (1.00, 1.01)
Female	1.04 (1.03, 1.04)	1.02 (1.01, 1.03)	1.06 (1.05, 1.06)	1.00 (0.99, 1.00)	1.03 (1.03, 1.04)	1.01 (1.00, 1.01)	1.02 (1.02, 1.03)
P for interaction	0.24	0.002	<0.001	<0.001	<0.001	<0.001	<0.001
Race							
White	1.03 (1.02, 1.03)	1.02 (1.01, 1.02)	1.05 (1.05, 1.06)	1.00 (0.99, 1.00)	1.03 (1.02, 1.03)	1.01 (1.01, 1.02)	1.02 (1.02, 1.03)
Black	1.10 (1.09, 1.12)	1.03 (1.01, 1.05)	1.00 (0.97, 1.02)	1.09 (1.07, 1.11)	1.08 (1.06, 1.09)	0.99 (0.98, 1.01)	1.01 (0.99, 1.03)
Other[a]	0.99 (0.98, 1.01)	0.94 (0.91, 0.96)	0.97 (0.93, 1.00)	0.91 (0.89, 0.93)	1.04 (1.03, 1.06)	0.93 (0.92, 0.95)	0.94 (0.92, 0.95)
P for interaction	<0.001	<0.001	<0.001	0.46	<0.001	<0.001	<0.001
Medicaid eligibility							
Non-dual eligible	1.01 (1.06, 1.02)	1.01 (1.00, 1.01)	1.03 (1.03, 1.04)	0.99 (0.98, 0.99)	1.02 (1.02, 1.03)	1.00 (1.00, 1.01)	1.01 (1.01, 1.02)
Dual-eligible	1.06 (1.05, 1.06)	1.02 (1.01, 1.03)	1.06 (1.05, 1.08)	1.00 (0.99, 1.01)	1.04 (1.03, 1.05)	1.00 (0.99, 1.01)	1.03 (1.02, 1.04)
P for interaction	0.03	<0.001	0.001	0.004	<0.001	<0.001	<0.001
Regions							
Midwest	1.05 (1.04, 1.07)	0.88 (0.86, 0.89)	1.01 (1.00, 1.03)	0.90 (0.89, 0.91)	1.09 (1.08, 1.11)	1.14 (1.12, 1.16)	0.96 (0.94, 0.97)
Northeast	0.98 (0.96, 0.99)	0.93 (0.92, 0.94)	0.94 (0.92, 0.96)	0.94 (0.93, 0.96)	1.17 (1.14, 1.20)	1.04 (1.02, 1.07)	0.97 (0.96, 0.98)
Southeast	1.06 (1.05, 1.07)	1.01 (1.00, 1.03)	0.92 (0.90, 0.94)	1.12 (1.11, 1.14)	1.09 (1.07, 1.10)	0.98 (0.98, 0.99)	1.02 (1.00, 1.03)
Southwest	1.00 (0.99, 1.02)	1.20 (1.17, 1.23)	1.25 (1.22, 1.28)	1.20 (1.17, 1.23)	0.93 (0.92, 0.94)	1.00 (0.99, 1.01)	1.08 (1.06, 1.10)

West	1.02 (1.02, 1.03)	1.07 (1.06, 1.09)	1.30 (1.26, 1.33)	1.00 (0.99, 1.01)	1.04 (1.03, 1.05)	1.00 (0.99, 1.01)	1.02 (1.01, 1.03)
P for interaction	0.44	<0.001	<0.001	<0.001	<0.001	0.04	<0.001

Note: IQR, interquartile range; $PM_{2.5}$, fine particulate matter.

[a]Other included Asian, Hispanic, American Indian, or Alaskan Native, and unknown.

P for interaction was assessed by including interaction terms between these potential modifiers and air pollutants.

All exposures were treated as 5-year-before window moving means of air pollutants.

Hazard ratios were calculated using the same interquartile ranges (IQR) as the main analysis.

This analysis was performed with a full cohort including 23,696,223 Medicare beneficiaries with 145,477,746 million person-years.

Cox proportional hazards models were used to estimate the hazard ratios. All models were adjusted for individual-level characteristics (age, sex, race, Medicaid eligibility), neighborhood-level socioeconomic indicators, calendar year, and indicator variables for the region.

© 2025 Deng Y et al. *JAMA Network Open*.

eTable 5. Hazard ratios and 95% CIs of per IQR increase in $PM_{2.5}$ major components associated with depression from single-pollutant models and multi-pollutant models, among the cohort with 5-year clean period.

	Model 1	Model 2	Model 3	Model 4
Elemental carbon	1.03 (1.03, 1.04)	1.04 (1.03, 1.05)	1.04 (1.04, 1.05)	1.03 (1.03, 1.04)
Ammonium	1.01 (1.01, 1.02)	-	1.04 (1.04, 1.05)	-
Sulfate	1.05 (1.04, 1.06)	1.07 (1.06, 1.08)	-	1.06 (1.05, 1.07)
Nitrate	0.99 (0.99, 1.00)	0.96 (0.96, 0.97)	0.95 (0.94, 0.96)	0.98 (0.97, 0.99)
Soil dust	1.03 (1.03, 1.04)	1.03 (1.02, 1.04)	1.02 (1.02, 1.03)	1.04 (1.03, 1.04)
Organic carbon	1.00 (1.00, 1.01)	0.98 (0.97, 0.99)	0.98 (0.98, 0.99)	0.99 (0.99, 1.00)
$PM_{2.5}$ mass	1.02 (1.01, 1.02)	-	-	-

Note: CI, confidence intervals; HR, Hazard ratios; IQR, interquartile range; $PM_{2.5}$, fine particulate matter.

Model 1: Single-pollutant model.

Model 2: Multi-pollutant model, including elemental carbon, sulfate, nitrate, organic carbon, and soil dust in one model simultaneously.

Model 3: Multi-pollutant model, including elemental carbon, ammonium, nitrate, organic carbon, and soil dust in one model simultaneously.

Model 4: Single-pollutant model with adjustment of the residual (i.e., subtracting the component of interest from total $PM_{2.5}$ mass). Ammonium has strong positive correlations with its residuals (r=0.81).

This analysis was performed with a full cohort including 23,696,223 Medicare beneficiaries with 145,477,746 million person-years.

Cox proportional hazards models were used to estimate the hazard ratios. All models were adjusted for individual-level characteristics (age, sex, race, Medicaid eligibility), neighborhood-level socioeconomic indicators, calendar year, and indicator variables for the region.

Hazard ratios were calculated using the same interquartile ranges (IQR) as the main analysis.

© 2025 Deng Y et al. *JAMA Network Open*.

eTable 6. Associations between $PM_{2.5}$ major components and depression estimated by quantile g-computation model among the cohort with 5-year clean period in the main and sensitivity analyses.

Air pollutants	Weights	Estimate (β)	HR (95% CI)
**Model 1**			
$PM_{2.5}$ mixtures		0.06	1.07 (1.06, 1.07)
Elemental carbon	0.25	0.02	
Ammonium	-0.67	-0.01	
Sulfate	0.34	0.03	
Nitrate	-0.05	-0.001	
Soil dust	0.41	0.04	
Organic carbon	-0.28	-0.01	
**Model 2**			
$PM_{2.5}$ mixtures		0.06	1.07 (1.06, 1.07)
Elemental carbon	0.28	0.02	
Ammonium			
Sulfate	0.27	0.02	
Nitrate	-0.48	-0.01	
Soil dust	0.46	0.04	
Organic carbon	-0.52	-0.01	
**Model 3**			
$PM_{2.5}$ mixtures		0.05	1.06 (1.05, 1.06)
Elemental carbon	0.37	0.02	
Ammonium	0.07	0.00	
Sulfate			
Nitrate	-0.53	-0.01	
Soil dust	0.56	0.04	
Organic carbon	-0.47	0.00	
**Model 4**			
$PM_{2.5}$ mixtures		0.07	1.07 (1.06, 1.08)
Elemental carbon	0.24	0.02	
Ammonium	-0.66	-0.02	
Sulfate	0.31	0.03	
Nitrate	-0.03	-0.001	
Soil dust	0.39	0.04	
Organic carbon	-0.31	-0.01	
Other $PM_{2.5}$ components	0.06	0.01	

© 2025 Deng Y et al. *JAMA Network Open.*

Note: CI, confidence intervals; HR, Hazard ratios; $PM_{2.5}$, fine particulate matter.

Model 1 includes six $PM_{2.5}$ major components in one model simultaneously.

Model 2 includes elemental carbon, sulfate, nitrate, organic carbon, and soil dust in one model simultaneously.

Model 3 includes elemental carbon, ammonium, nitrate, organic carbon, and soil dust in one model simultaneously.

Model 4 includes six $PM_{2.5}$ major components and other $PM_{2.5}$ components (total $PM_{2.5}$ mass minus the sum of six key components) in one model simultaneously.

This analysis was performed with a full cohort including 23,696,223 Medicare beneficiaries with 145,477,746 million person-years.

Quantile g-computation was used to estimate the joint effects of the $PM_{2.5}$ major component mixtures on incident depression risk.

All models were adjusted for individual-level characteristics (age, sex, race, Medicaid eligibility), neighborhood-level socioeconomic indicators, calendar year, and indicator variables for the region.

© 2025 Deng Y et al. *JAMA Network Open*.

eTable 7. Hazard ratios and 95% CIs of per IQR increase in $PM_{2.5}$ major components associated with depression using different exposure data from single-pollutant models among the cohort with 5-year clean period.

	Exposure I[a]	Exposure II[b]
Elemental carbon	1.03 (1.03, 1.04)	1.01 (1.00, 1.01)
Ammonium	1.01 (1.01, 1.02)	1.01 (1.00, 1.02)
Sulfate	1.05 (1.04, 1.06)	1.04 (1.03, 1.05)
Nitrate	0.99 (0.99, 1.00)	0.98 (0.98, 0.99)
Soil dust	1.03 (1.03, 1.04)	1.02 (1.02, 1.02)
Organic carbon	1.00 (1.00, 1.01)	1.01 (1.01, 1.02)
$PM_{2.5}$ mass	1.02 (1.01, 1.02)	1.02 (1.01, 1.02)

Note: CI, confidence intervals; HR, Hazard ratios; IQR, interquartile range; $PM_{2.5}$, fine particulate matter.

[a]Main analysis: Exposure I pollutants were derived from Amini et al.[20]

[b]Exposure II pollutants were derived from van Donkelaar et al.[15]

This analysis was performed with a full cohort including 23,696,223 Medicare beneficiaries with 145,477,746 million person-years.

Cox proportional hazards models were used to estimate the hazard ratios. All models were adjusted for individual-level characteristics (age, sex, race, Medicaid eligibility), neighborhood-level socioeconomic indicators, calendar year, and indicator variables for the region.

Hazard ratios were calculated using the same interquartile ranges (IQR) as the main analysis.

© 2025 Deng Y et al. *JAMA Network Open*.

eTable 8. Hazard ratios and 95% CIs of per IQR increase in $PM_{2.5}$ major components associated with depression using varying clean periods from single-pollutant model.

	Cohort with 5-year clean period	Cohort with 3-year clean period	Cohort with 10-year clean period
Per IQR increase			
Elemental carbon	1.03 (1.03, 1.04)	1.03 (1.02, 1.03)	1.05 (1.04, 1.05)
Ammonium	1.01 (1.01, 1.02)	1.01 (1.00, 1.02)	1.04 (1.03, 1.05)
Sulfate	1.05 (1.04, 1.06)	1.04 (1.03, 1.05)	1.11 (1.10, 1.12)
Nitrate	0.99 (0.99, 1.00)	0.99 (0.99, 1.00)	1.02 (1.01, 1.03)
Soil dust	1.03 (1.03, 1.04)	1.03 (1.03, 1.04)	1.04 (1.03, 1.04)
Organic carbon	1.00 (1.00, 1.01)	1.00 (1.00, 1.01)	1.00 (1.00, 1.01)
$PM_{2.5}$ mass	1.02 (1.01, 1.02)	1.01 (1.01, 1.02)	1.03 (1.02, 1.03)

Note: CI, confidence intervals; IQR, interquartile range; $PM_{2.5}$, fine particulate matter.

Cohort with 5-year clean period was performed with a full cohort including 23,696,223 Medicare beneficiaries with 145,477,746 million person-years.

Cohort with 3-year clean period was performed with a full cohort including 30,582,355 Medicare beneficiaries with 203,046,129 million person-years.

Cohort with 10-year clean period was performed with a full cohort including 11,362,663 Medicare beneficiaries with 55,194,780 million person-years.

Cox proportional hazards models were used to estimate the hazard ratios. All models were adjusted for individual-level characteristics (age, sex, race, Medicaid eligibility), neighborhood-level socioeconomic indicators, calendar year, and indicator variables for the region.

Hazard ratios were calculated using the same interquartile ranges (IQR) as the main analysis.

© 2025 Deng Y et al. *JAMA Network Open*.

eTable 9. Hazard ratios and 95% CIs of per IQR increase in $PM_{2.5}$ major components associated with depression using varying lag periods from single-pollutant models among the cohort with 5-year clean period.

	Average [a](Lag 1-5)	Lag 0	Lag 1	Average [b](Lag 1-3)
Elemental carbon	1.03 (1.03, 1.04)	1.03 (1.03, 1.04)	1.03 (1.02, 1.04)	1.03 (1.03, 1.04)
Ammonium	1.01 (1.01, 1.02)	1.02 (1.01, 1.03)	1.01 (1.01, 1.02)	1.01 (1.01, 1.02)
Sulfate	1.05 (1.04, 1.06)	1.04 (1.03, 1.05)	1.04 (1.03, 1.05)	1.05 (1.04, 1.06)
Nitrate	0.99 (0.99, 1.00)	1.01 (1.00, 1.02)	1.00 (0.99, 1.01)	1.00 (0.99, 1.00)
Soil dust	1.03 (1.03, 1.04)	1.02 (1.01, 1.02)	1.03 (1.02, 1.03)	1.03 (1.03, 1.04)
Organic carbon	1.00 (1.00, 1.01)	1.00 (1.00, 1.01)	1.00 (1.00, 1.00)	1.00 (1.00, 1.01)
$PM_{2.5}$ mass	1.02 (1.01, 1.02)	1.03 (1.02, 1.03)	1.02 (1.01, 1.02)	1.02 (1.01, 1.02)

Note: CI, confidence intervals; IQR, interquartile range; $PM_{2.5}$, fine particulate matter.

[a]All exposures were treated as 5-year-before window moving means of air pollutants.

[b]All exposures were treated as 3-year-before window moving means of air pollutants.

Hazard ratios were calculated using the same interquartile ranges (IQR) as the main analysis.

This analysis was performed with a full cohort including 23,696,223 Medicare beneficiaries with 145,477,746 million person-years.

Cox proportional hazards models were used to estimate the hazard ratios. All models were adjusted for individual-level characteristics (age, sex, race, Medicaid eligibility), neighborhood-level socioeconomic indicators, calendar year, and indicator variables for the region.

© 2025 Deng Y et al. *JAMA Network Open*.

eTable 10. Hazard ratios and 95% CIs of per IQR increase in $PM_{2.5}$ major components associated with depression from single-pollutant models among the cohort with 5-year clean period, derived from the linear rate models.

	Cox model	Linear rate model
Elemental carbon	1.03 (1.03, 1.04)	1.07 (1.07, 1.08)
Ammonium	1.01 (1.01, 1.02)	1.07 (1.07, 1.08)
Sulfate	1.05 (1.04, 1.06)	1.13 (1.12, 1.14)
Nitrate	0.99 (0.99, 1.00)	1.04 (1.04, 1.05)
Soil dust	1.03 (1.03, 1.04)	1.04 (1.03, 1.05)
Organic carbon	1.00 (1.00, 1.01)	1.02 (1.01, 1.02)
$PM_{2.5}$ mass	1.02 (1.01, 1.02)	1.05 (1.04, 1.05)

Note: CI, confidence intervals; IQR, interquartile range; $PM_{2.5}$, fine particulate matter.

This analysis was performed with a full cohort including 23,696,223 Medicare beneficiaries with 145,477,746 million person-years.

All models were adjusted for individual-level characteristics (age, sex, race, Medicaid eligibility), neighborhood-level socioeconomic indicators, calendar year, and indicator variables for the region.

Hazard ratios were calculated using the same interquartile ranges (IQR) as the main analysis.

Linear regression models for the rate of depression (events/person-time) with a generalized estimating equation. The outcome is rate, and the coefficient of exposure is the incremental probability of the event (i.e., the increase in rate) for a unit/IQR change in exposure. The baseline incidence rate of depression was calculated based on the number of events and total person years in Table 1.

© 2025 Deng Y et al. *JAMA Network Open*.

eTable 11. Hazard ratios and 95% CIs of IQR increase in $PM_{2.5}$ major components associated with depression from single-pollutant models among the cohort with 5-year clean period, accounting for potential outcome misclassification via adjusting data for assumed sensitivity and specificity of classification.

	Cox model	Corrected hazard ratio from Cox model
Elemental carbon	1.03 (1.03, 1.04)	1.15 (1.14, 1.15)
Ammonium	1.01 (1.01, 1.02)	1.04 (1.04, 1.05)
Sulfate	1.05 (1.04, 1.06)	1.14 (1.13, 1.15)
Nitrate	0.99 (0.99, 1.00)	0.97 (0.97, 0.98)
Soil dust	1.03 (1.03, 1.04)	0.97 (0.97, 0.98)
Organic carbon	1.00 (1.00, 1.01)	1.00 (0.99, 1.00)
$PM_{2.5}$ mass	1.02 (1.01, 1.02)	1.09 (1.08, 1.09)

Note: CI, confidence intervals; IQR, interquartile range; $PM_{2.5}$, fine particulate matter.

This analysis was performed with a full cohort including 23,696,223 Medicare beneficiaries with 145,477,746 million person-years.

All models were adjusted for individual-level characteristics (age, sex, race, Medicaid eligibility), neighborhood-level socioeconomic indicators, calendar year, and indicator variables for the region.

Hazard ratios were calculated using the same interquartile ranges (IQR) as the main analysis.

This analysis consider the possible effect of outcome misclassification following methods similar to those described by Fox et al.[17] and adjusting the observed outcomes for each stratum based on the estimates of Medicare sensitivity and specificity from Hwang et al.[18] to estimate the expected true number of cases.

© 2025 Deng Y et al. *JAMA Network Open*.

eReferences

1. Di Q, Amini H, Shi L, et al. An ensemble-based model of PM(2.5) concentration across the contiguous United States with high spatiotemporal resolution. *Environ Int*. 2019;130:104909. doi:10.1016/j.envint.2019.104909
2. Qiu X, Shi L, Kubzansky LD, et al. Association of long-term exposure to air pollution with late-life depression in older adults in the US. *Jama Netw Open*. 2023;6(2):e2253668. doi:10.1001/jamanetworkopen.2022.53668
3. Di Q, Wang Y, Zanobetti A, et al. Air pollution and mortality in the Medicare population. *New Engl J Med*. 2017;376(26):2513-2522. doi:10.1056/NEJMoa1702747
4. Lee W, Wu X, Heo S, et al. Air pollution and acute kidney injury in the U.S. Medicare population: A longitudinal cohort study. *Environ Health Persp*. 2023;131(4):47008. doi:10.1289/EHP10729
5. Census U. American Community Survey 1-Year Data (2005–2019), https://www.census.gov/data/developers/data-sets/acs-1year.html. 2020
6. Census U. Summary File 3 Dataset, https://www.census.gov/data/datasets/2000/dec/summary-file-3.html. 2002
7. Census U. Summary File 1 Dataset, https://www.census.gov/data/datasets/2010/dec/summary-file-1.html. 2011
8. CDC U. Behavioral Risk Factor Surveillance System https://www.cdc.gov/brfss/annual_data/annual_data.html. 2020
9. Area Health Resources Files, https://data.hrsa.gov/topics/health-workforce/ahrf. 2019. doi:10.1007/s11356-021-12357-3
10. Junninen H, Niska H, Tuppurainen K, Ruuskanen J, Kolehmainen M. Methods for imputation of missing values in air quality data sets. *Atmos Environ*.

2004;38(18):2895-2907. doi:10.1016/j.atmosenv.2004.02.026

11. Shi L, Steenland K, Li H, et al. A national cohort study (2000-2018) of long-term air pollution exposure and incident dementia in older adults in the United States. *Nat Commun*. 2021;12(1):6754. doi:10.1038/s41467-021-27049-2

12. Shi L, Zhu Q, Wang Y, et al. Incident dementia and long-term exposure to constituents of fine particle air pollution: A national cohort study in the United States. *P Natl Acad Sci Usa*. 2023;120(1):e2211282119. doi:10.1073/pnas.2211282119

13. Kaufman JS, MacLehose RF. Which of these things is not like the others? *Cancer-Am Cancer Soc*. 2013;119(24):4216-4222. doi:10.1002/cncr.28359

14. Chronic Conditions Data Warehouse. Condition categories. [. https://www2.ccwdata.org/web/guest/condition-categories.

15. van Donkelaar A, Martin RV, Li C, Burnett RT. Regional estimates of chemical composition of fine particulate matter using a combined geoscience-statistical method with information from satellites, models, and monitors. *Environ Sci Technol*. 2019;53(5):2595-2611. doi:10.1021/acs.est.8b06392

16. Hutcheon JA, Chiolero A, Hanley JA. Random measurement error and regression dilution bias. *Bmj-Brit Med J*. 2010;340:c2289. doi:10.1136/bmj.c2289

17. Fox MP, Lash TL, Greenland S. A method to automate probabilistic sensitivity analyses of misclassified binary variables. *Int J Epidemiol*. 2005;34(6):1370-6. doi:10.1093/ije/dyi184

18. Hwang S, Jayadevappa R, Zee J, et al. Concordance between clinical diagnosis and medicare claims of depression among older primary care patients. *Am J Geriat Psychiat*. 2015;23(7):726-34. doi:10.1016/j.jagp.2014.08.009

19. Shiraki R, Holmen BA. Airborne respirable silica near a sand and gravel facility in

© 2025 Deng Y et al. *JAMA Network Open*.

central California: XRD and elemental analysis to distinguish source and background quartz. *Environ Sci Technol*. 2002;36(23):4956-61. doi:10.1021/es0257265

20. Amini H, Danesh-Yazdi M, Di Q, et al. Hyperlocal super-learned $PM_{2.5}$ components across the contiguous US. *Research Square*. 2022;57(1):96-108. doi:10.1021/acs.est.2c08096

© 2025 Deng Y et al. *JAMA Network Open*.
