# Supplementary material for: Exposure to Multiple Fine Particulate Matter Components and Incident Depression in the US Medicare Population
Source: JAMA Netw Open. 2025 Dec 22;8(12):e2551042. doi: 10.1001/jamanetworkopen.2025.51042 (PMC12723547; doi:10.1001/jamanetworkopen.2025.51042)
Supplement: Supplement 2. — Data Sharing Statement [file jamanetwopen-e2551042-s002.pdf]

Data Sharing Statement

Deng. Exposure to Multiple $PM_{2.5}$ Major Components and Incident Depression in the US Medicare Population. *JAMA Netw Open*. Published December 22, 2025. doi:10.1001/jamanetworkopen.2025.51042

Data
Data available: No
